# 3,3′-Dibenzyl-1,1′-ethyl­enediimidazolium dibromide

**DOI:** 10.1107/S1600536810031934

**Published:** 2010-08-18

**Authors:** Hon Man Lee, Pi-Yun Cheng

**Affiliations:** a National Changhua University of Education, Department of Chemistry, Changhua, Taiwan 50058

## Abstract

In the title compound, C_22_H_24_N_4_^2+^·2Br^−^, the imidazolium dication is located on a crystallographic inversion center. The imidazole and benzene rings make a dihedral angle of 73.1 (9)°. In the crystal, non-classical inter­molecular C—H⋯Br hydrogen bonds link the ion pairs into a two-dimensional network.

## Related literature

For related structures of bis­(imidazolium) salts, see: Baker *et al.* (2002 ▶); Lee *et al.* (2004 ▶, 2007 ▶, 2008 ▶); Jin *et al.* (2007 ▶); Lee & Lu (2008 ▶); Paulose *et al.* (2008 ▶) and of methyl­ene-linked bis­(imidazolium) salts, see: Cheng *et al.* (2006 ▶); Lee & Chiu (2004 ▶); Lee *et al.* (2004 ▶).
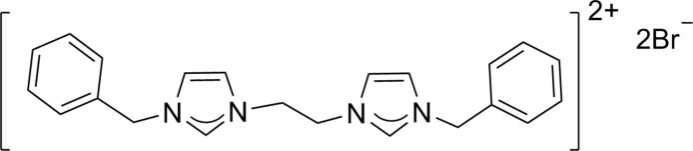


## Experimental

### 

#### Crystal data

C_22_H_24_N_4_^2+^·2Br^−^
*M* *_r_* = 504.27
Monoclinic, 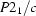

*a* = 16.4674 (8) Å
*b* = 5.2099 (2) Å
*c* = 12.3736 (6) Å
β = 96.495 (3)°
*V* = 1054.76 (8) Å^3^
*Z* = 2
Mo *K*α radiation
μ = 3.86 mm^−1^
*T* = 150 K
0.25 × 0.20 × 0.20 mm

#### Data collection

Bruker SMART APEXII diffractometer
Absorption correction: multi-scan (*SADABS*; Sheldrick, 2003 ▶) *T*_min_ = 0.446, *T*_max_ = 0.513
10232 measured reflections
2542 independent reflections
1915 reflections with *I* > 2σ
*R*_int_ = 0.047

#### Refinement

*R*[*F* ^2^ > 2σ(*F* ^2^)] = 0.031
*wR*(*F* ^2^) = 0.074
*S* = 1.03
2542 reflections
127 parameters
H-atom parameters constrained
Δρ_max_ = 0.49 e Å^−3^
Δρ_min_ = −0.46 e Å^−3^

### 

Data collection: *APEX2* (Bruker, 2007 ▶); cell refinement: *SAINT* (Bruker, 2007 ▶); data reduction: *SAINT*; program(s) used to solve structure: *SHELXTL* (Sheldrick, 2008 ▶); program(s) used to refine structure: *SHELXTL*; molecular graphics: *SHELXTL*; software used to prepare material for publication: *DIAMOND* (Brandenburg, 2006 ▶).

## Supplementary Material

Crystal structure: contains datablocks I, global. DOI: 10.1107/S1600536810031934/om2353sup1.cif

Structure factors: contains datablocks I. DOI: 10.1107/S1600536810031934/om2353Isup2.hkl

Additional supplementary materials:  crystallographic information; 3D view; checkCIF report

## Figures and Tables

**Table 1:** Hydrogen-bond geometry (Å, °)

*D*—H⋯*A*	*D*—H	H⋯*A*	*D*⋯*A*	*D*—H⋯*A*
C1—H1⋯Br1^i^	0.95	2.86	3.663 (2)	143
C3—H3⋯Br1^ii^	0.95	2.77	3.683 (2)	162
C4—H4*B*⋯Br1^i^	0.99	2.83	3.661 (3)	142

Symmetry codes: (i) 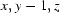; (ii) 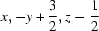.

**Fig. 1.**
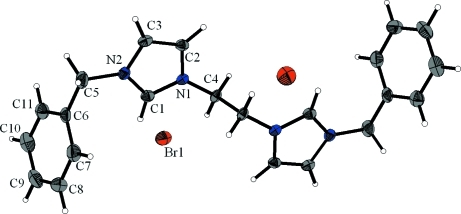
The structure of the title compound, showing 50% probability displacement ellipsoids for the non-hydrogen atoms. The H atoms are depicted by circles of an arbitrary radius. The unlabelled atoms of the imidazolium cation and the bromide anion are related to the labelled ones by 1 - x, -y, 1 - z.

**Fig. 2.**
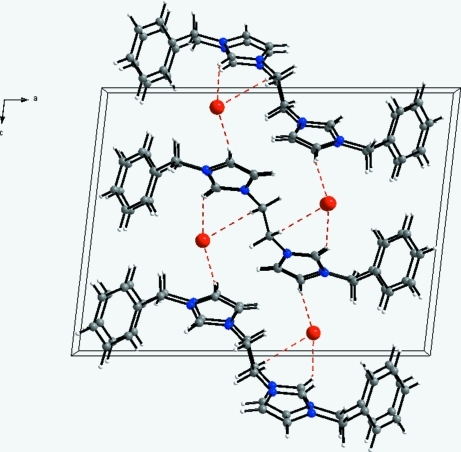
A view of the crystal packing along the b axis. Hydrogen bonds are shown as dashed lines.

## supplementary crystallographic information

### Comment

The title compound is a precursor to the formation of bidentate *N*-heterocyclic carbene ligand (Lee *et al.* 2004). It can be easily prepared by the reaction of 1,2-dibromoethane and 1-benzylimidazole in tetrahydrofuran (Lee *et al.* 2004).

The dication is located on a crystallographic inversion center. The non-classical intermolecular hydrogen bonds of the type C—H···Br link the imidazolium dications and bromide anions into a two dimensional hydrogen bonded network.

Ethylene-linked bis(imidazolium) salts similar to the title compound were reported by us (Lee *et al.*, 2004; Lee *et al.* 2007; Lee & Lu (2008); Lee *et al.* 2008) and others (Baker *et al.* 2002; Jin *et al.* 2007; Paulose *et al.* 2008). We also published crystal structures of relevant methylene-linked bis(imidazolium) salts (Lee *et al.*, 2004; Lee & Chiu, 2004; Cheng *et al.* 2006).

### Experimental

The compound was prepared according to the literature procedure (Lee *et al.*, 2004). Suitable crystals were obtained by slow diffusion of diethyl ether into a DMF solution of the compound at room temperature.

### Refinement

All the hydrogen atoms were discerned in the difference Fourier map, nevertheless, all the H atoms were positioned geometrically and refined as riding atoms, with C_aryl_—H = 0.95, C_methylene_ —H = 0.99 Å while *U*_iso_(H) = 1.2*U*_eq_(C) for all the H atoms.

### Crystal data

**Table:**

C_22_H_24_N_4_^2+^·2Br^−^	*F*(000) = 508
*M**_r_* = 504.27	*D*_x_ = 1.588 Mg m^−^^3^
Monoclinic, *P*2_1_/*c*	Mo *K*α radiation, λ = 0.71073 Å
Hall symbol: -P 2ybc	Cell parameters from 2242 reflections
*a* = 16.4674 (8) Å	θ = 2.5–24.5°
*b* = 5.2099 (2) Å	µ = 3.86 mm^−^^1^
*c* = 12.3736 (6) Å	*T* = 150 K
β = 96.495 (3)°	Block, colorless
*V* = 1054.76 (8) Å^3^	0.25 × 0.20 × 0.20 mm
*Z* = 2	

### Data collection

**Table:**

Bruker SMART APEXII diffractometer	2542 independent reflections
Radiation source: fine-focus sealed tube	1915 reflections with *I* > 2σ
graphite	*R*_int_ = 0.047
ω scans	θ_max_ = 28.0°, θ_min_ = 2.5°
Absorption correction: multi-scan (*SADABS*; Sheldrick, 2003)	*h* = −21→21
*T*_min_ = 0.446, *T*_max_ = 0.513	*k* = −6→6
10232 measured reflections	*l* = −16→14

### Refinement

**Table:**

Refinement on *F*^2^	Primary atom site location: structure-invariant direct methods
Least-squares matrix: full	Secondary atom site location: difference Fourier map
*R*[*F*^2^ > 2σ(*F*^2^)] = 0.031	Hydrogen site location: inferred from neighbouring sites
*wR*(*F*^2^) = 0.074	H-atom parameters constrained
*S* = 1.03	*w* = 1/[σ^2^(*F*_o_^2^) + (0.0338*P*)^2^ + 0.006*P*] where *P* = (*F*_o_^2^ + 2*F*_c_^2^)/3
2542 reflections	(Δ/σ)_max_ = 0.001
127 parameters	Δρ_max_ = 0.49 e Å^−^^3^
0 restraints	Δρ_min_ = −0.46 e Å^−^^3^

### Special details

**Table:**

Geometry. All e.s.d.'s (except the e.s.d. in the dihedral angle between two l.s. planes) are estimated using the full covariance matrix. The cell e.s.d.'s are taken into account individually in the estimation of e.s.d.'s in distances, angles and torsion angles; correlations between e.s.d.'s in cell parameters are only used when they are defined by crystal symmetry. An approximate (isotropic) treatment of cell e.s.d.'s is used for estimating e.s.d.'s involving l.s. planes.
Refinement. Refinement of *F*^2^ against ALL reflections. The weighted *R*-factor *wR* and goodness of fit *S* are based on *F*^2^, conventional *R*-factors *R* are based on *F*, with *F* set to zero for negative *F*^2^. The threshold expression of *F*^2^ > σ(*F*^2^) is used only for calculating *R*-factors(gt) *etc*. and is not relevant to the choice of reflections for refinement. *R*-factors based on *F*^2^ are statistically about twice as large as those based on *F*, and *R*- factors based on ALL data will be even larger.

### Fractional atomic coordinates and isotropic or equivalent isotropic displacement parameters (Å^2^)

**Table:**

	*x*	*y*	*z*	*U*_iso_*/*U*_eq_	
Br1	0.326856 (16)	0.58076 (5)	0.570787 (19)	0.02456 (10)	
C1	0.34814 (15)	0.0860 (5)	0.37001 (19)	0.0191 (5)	
H1	0.3171	−0.0415	0.4018	0.023*	
C2	0.45155 (15)	0.3145 (5)	0.32355 (19)	0.0186 (5)	
H2	0.5056	0.3725	0.3176	0.022*	
C3	0.38199 (15)	0.4239 (5)	0.27857 (19)	0.0196 (5)	
H3	0.3777	0.5737	0.2345	0.024*	
C4	0.48543 (14)	−0.0683 (4)	0.44729 (18)	0.0173 (5)	
H4A	0.5327	−0.1128	0.4082	0.021*	
H4B	0.4569	−0.2290	0.4628	0.021*	
C5	0.23178 (16)	0.3432 (5)	0.2752 (2)	0.0300 (7)	
H5A	0.2227	0.3566	0.1949	0.036*	
H5B	0.2199	0.5131	0.3056	0.036*	
C6	0.17344 (15)	0.1494 (5)	0.3122 (2)	0.0230 (6)	
C7	0.15780 (16)	0.1430 (6)	0.4197 (2)	0.0324 (7)	
H7	0.1842	0.2621	0.4701	0.039*	
C8	0.10409 (18)	−0.0349 (6)	0.4545 (3)	0.0381 (8)	
H8	0.0944	−0.0402	0.5287	0.046*	
C9	0.06422 (16)	−0.2064 (6)	0.3799 (3)	0.0380 (8)	
H9	0.0271	−0.3289	0.4031	0.046*	
C10	0.07858 (17)	−0.1982 (6)	0.2731 (3)	0.0350 (7)	
H10	0.0508	−0.3134	0.2221	0.042*	
C11	0.13354 (16)	−0.0224 (5)	0.2392 (2)	0.0281 (6)	
H11	0.1439	−0.0199	0.1652	0.034*	
N1	0.42965 (12)	0.1019 (4)	0.37997 (15)	0.0166 (4)	
N2	0.31810 (11)	0.2793 (4)	0.30799 (15)	0.0178 (4)	

### Atomic displacement parameters (Å^2^)

**Table:**

	*U*^11^	*U*^22^	*U*^33^	*U*^12^	*U*^13^	*U*^23^
Br1	0.03007 (16)	0.02334 (15)	0.02017 (15)	−0.00608 (12)	0.00241 (10)	−0.00064 (12)
C1	0.0225 (13)	0.0160 (13)	0.0189 (12)	−0.0020 (10)	0.0028 (10)	0.0006 (11)
C2	0.0212 (14)	0.0178 (12)	0.0172 (12)	−0.0042 (10)	0.0035 (10)	−0.0002 (10)
C3	0.0263 (14)	0.0179 (13)	0.0148 (12)	−0.0014 (11)	0.0030 (10)	0.0003 (11)
C4	0.0188 (12)	0.0161 (12)	0.0162 (12)	0.0047 (10)	−0.0014 (9)	0.0011 (10)
C5	0.0204 (14)	0.0286 (15)	0.0395 (17)	0.0048 (11)	−0.0040 (12)	0.0103 (12)
C6	0.0147 (13)	0.0236 (14)	0.0298 (15)	0.0041 (10)	−0.0025 (11)	0.0049 (11)
C7	0.0248 (16)	0.0372 (17)	0.0338 (17)	−0.0022 (11)	−0.0022 (13)	−0.0028 (13)
C8	0.0304 (17)	0.053 (2)	0.0318 (17)	0.0014 (14)	0.0078 (13)	0.0109 (15)
C9	0.0200 (15)	0.0320 (17)	0.062 (2)	−0.0003 (12)	0.0056 (14)	0.0132 (16)
C10	0.0263 (16)	0.0279 (16)	0.050 (2)	0.0014 (12)	0.0011 (14)	−0.0091 (14)
C11	0.0241 (15)	0.0290 (15)	0.0310 (16)	0.0058 (11)	0.0023 (12)	−0.0036 (12)
N1	0.0205 (11)	0.0154 (10)	0.0135 (10)	0.0004 (8)	0.0012 (8)	0.0004 (8)
N2	0.0193 (11)	0.0155 (11)	0.0179 (11)	0.0009 (8)	−0.0008 (8)	0.0020 (9)

### Geometric parameters (Å, °)

**Table:**

C1—N2	1.328 (3)	C5—H5A	0.9900
C1—N1	1.336 (3)	C5—H5B	0.9900
C1—H1	0.9500	C6—C7	1.384 (4)
C2—C3	1.343 (3)	C6—C11	1.384 (4)
C2—N1	1.379 (3)	C7—C8	1.383 (4)
C2—H2	0.9500	C7—H7	0.9500
C3—N2	1.376 (3)	C8—C9	1.394 (4)
C3—H3	0.9500	C8—H8	0.9500
C4—N1	1.467 (3)	C9—C10	1.369 (4)
C4—C4^i^	1.515 (4)	C9—H9	0.9500
C4—H4A	0.9900	C10—C11	1.386 (4)
C4—H4B	0.9900	C10—H10	0.9500
C5—N2	1.472 (3)	C11—H11	0.9500
C5—C6	1.501 (4)		
			
N2—C1—N1	108.1 (2)	C11—C6—C5	120.6 (3)
N2—C1—H1	125.9	C6—C7—C8	120.7 (3)
N1—C1—H1	125.9	C6—C7—H7	119.7
C3—C2—N1	106.9 (2)	C8—C7—H7	119.7
C3—C2—H2	126.5	C7—C8—C9	119.6 (3)
N1—C2—H2	126.5	C7—C8—H8	120.2
C2—C3—N2	107.5 (2)	C9—C8—H8	120.2
C2—C3—H3	126.3	C10—C9—C8	119.9 (3)
N2—C3—H3	126.3	C10—C9—H9	120.0
N1—C4—C4^i^	108.8 (2)	C8—C9—H9	120.0
N1—C4—H4A	109.9	C9—C10—C11	120.2 (3)
C4^i^—C4—H4A	109.9	C9—C10—H10	119.9
N1—C4—H4B	109.9	C11—C10—H10	119.9
C4^i^—C4—H4B	109.9	C6—C11—C10	120.6 (3)
H4A—C4—H4B	108.3	C6—C11—H11	119.7
N2—C5—C6	113.3 (2)	C10—C11—H11	119.7
N2—C5—H5A	108.9	C1—N1—C2	108.7 (2)
C6—C5—H5A	108.9	C1—N1—C4	125.0 (2)
N2—C5—H5B	108.9	C2—N1—C4	126.2 (2)
C6—C5—H5B	108.9	C1—N2—C3	108.8 (2)
H5A—C5—H5B	107.7	C1—N2—C5	128.0 (2)
C7—C6—C11	119.1 (3)	C3—N2—C5	123.1 (2)
C7—C6—C5	120.3 (2)		
			
N1—C2—C3—N2	0.3 (3)	N2—C1—N1—C4	176.4 (2)
N2—C5—C6—C7	76.1 (3)	C3—C2—N1—C1	−0.7 (3)
N2—C5—C6—C11	−105.0 (3)	C3—C2—N1—C4	−176.2 (2)
C11—C6—C7—C8	1.0 (4)	C4^i^—C4—N1—C1	−100.5 (3)
C5—C6—C7—C8	180.0 (3)	C4^i^—C4—N1—C2	74.3 (3)
C6—C7—C8—C9	−1.2 (4)	N1—C1—N2—C3	−0.7 (3)
C7—C8—C9—C10	0.2 (4)	N1—C1—N2—C5	−179.3 (2)
C8—C9—C10—C11	1.0 (4)	C2—C3—N2—C1	0.2 (3)
C7—C6—C11—C10	0.1 (4)	C2—C3—N2—C5	178.9 (2)
C5—C6—C11—C10	−178.8 (2)	C6—C5—N2—C1	−4.6 (4)
C9—C10—C11—C6	−1.1 (4)	C6—C5—N2—C3	176.9 (2)
N2—C1—N1—C2	0.9 (3)		

Symmetry codes: (i) −*x*+1, −*y*, −*z*+1.

### Hydrogen-bond geometry (Å, °)

**Table:**

*D*—H···*A*	*D*—H	H···*A*	*D*···*A*	*D*—H···*A*
C1—H1···Br1^ii^	0.95	2.86	3.663 (2)	143.
C3—H3···Br1^iii^	0.95	2.77	3.683 (2)	162.
C4—H4B···Br1^ii^	0.99	2.83	3.661 (3)	142.

Symmetry codes: (ii) *x*, *y*−1, *z*; (iii) *x*, −*y*+3/2, *z*−1/2.
